# Cross-seeding between Aβ and SEVI indicates a pathogenic link and gender difference between alzheimer diseases and AIDS

**DOI:** 10.1038/s42003-022-03343-7

**Published:** 2022-05-05

**Authors:** Yijing Tang, Dong Zhang, Yanxian Zhang, Yonglan Liu, Yifat Miller, Keven Gong, Jie Zheng

**Affiliations:** 1 Department of Chemical, Biomolecular, and Corrosion Engineering, The University of Akron, 44325 Akron, OH USA; 2 Department of Chemistry Ben-Gurion, University of the Negev, 84105 Be’er Sheva, Israel; 3 Western Reserve Academy, Hudson, 44236 OH USA

**Keywords:** Molecular conformation, Neurodegeneration

## Abstract

Amyloid-β (Aβ) and semen-derived enhancer of viral infection (SEVI) are considered as the two causative proteins for central pathogenic cause of Alzheimer’s disease (AD) and HIV/AIDS, respectively. Separately, Aβ-AD and SEVI-HIV/AIDS systems have been studied extensively both in fundamental research and in clinical trials. Despite significant differences between Aβ-AD and SEVI-HIV/AIDS systems, they share some commonalities on amyloid and antimicrobial characteristics between Aβ and SEVI, there are apparent overlaps in dysfunctional neurological symptoms between AD and HIV/AIDS. Few studies have reported a potential pathological link between Aβ-AD and SEVI-HIV/AIDS at a protein level. Here, we demonstrate the cross-seeding interactions between Aβ and SEVI proteins using in vitro and in vivo approaches. Cross-seeding of SEVI with Aβ enabled to completely prevent Aβ aggregation at sub-stoichiometric concentrations, disaggregate preformed Aβ fibrils, reduce Aβ-induced cell toxicity, and attenuate Aβ-accumulated paralysis in transgenic AD C. elegans. This work describes a potential crosstalk between AD and HIV/AIDS via the cross-seeding between Aβ and SEVI, identifies SEVI as Aβ inhibitor for possible treatment or prevention of AD, and explains the role of SEVI in the gender difference in AD.

## Abstract (web summary)

Cross-seeding of SEVI, a protein that enhances the infectivity of HIV virus, with Amyloid-β (Aβ) can prevent Aβ aggregation, disaggregate preformed Aβ fibrils, reduce Aβ-induced cell toxicity, and attenuate Aβ-accumulated paralysis in a transgenic C. elegans model of Alzheimer’s Disease.

## Introduction

Protein–protein interactions play an unquestionable role in virtually every cellular process in both healthy and diseased people^1^. A protein rarely acts alone to achieve its (dys)functions, instead more than 80% of all proteins achieve their (dys)functions by interacting with others. Different from homogeneous interactions between the same family of proteins associated with specific diseases, heterogeneous interactions between the two distinct families of disease-related proteins are far more complex for better understanding not only the pathological causes of each disease, but also molecular crosstalk between different human diseases.

**Fig. 1:**
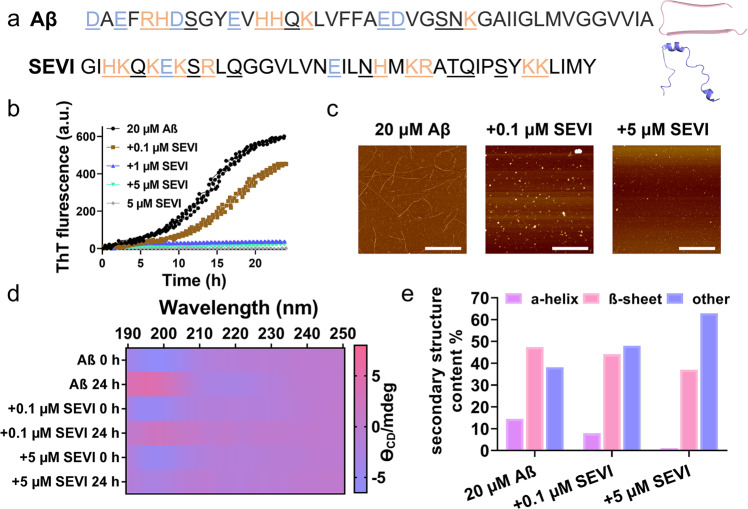
Cross-seeding of SEVI with Aβ to induce the inhibition effect on Aβ aggregation. **a** Sequence and structure of Aβ with a U-bent, β-structure and SEVI with a α-helix-structure. Color ID: polar residues (underline), non-polar residues (black), positively charged residues (orange), and negatively charged residues (blue). **b** Dose-dependent inhibition effect of SEVI (0.1–5 µM) on Aβ (20 µM) aggregation by ThT fluorescence assays. All data represent mean ± standard error of triplicate measurements. (*n* = 3). **c** AFM images of pure Aβ (20 µM) and cross-seeding Aβ-SEVI (20 µM:0.1–5 µM) systems at 24 h. Scale bars are 1 µm. **d** Time-dependent structure transition map of Aβ (20 µM) in the absence and presence of SEVI (0.1–5 µM) at 0 h and 24 h. **e** Final secondary structure distributions of pure Aβ (20 µM) and cross-seeding Aβ-SEVI (20 µM:0.1–5 µM) aggregates at 24 h.

Alzheimer’s disease (AD) and acquired immune deficiency syndrome (AIDS) are the two completely different diseases. AD is an age-dependent, neurodegenerative disorder^2,3^. Current prevailing “amyloid cascade hypothesis” postulates that the deposition of misfolded Aβ aggregates with highly ordered, β-sheet structures (namely amyloids) onto human brain is a pathological hallmark of AD^4–7^. Differently, AIDS is an infectious disease and mainly caused by HIV infection. SEVI (Semen-derived Enhancer of Viral Infection) - fragments of prostatic acid phosphatase (PAP_248–286_, a highly abundant protein found in human semen) - act as a very dramatic enhancer of HIV infectivity by the four to five orders of magnitude of HIV virus-cell attachment and fusion in multiple viral and host cell genotypes^8,9^. Evidently, Aβ and SEVI proteins are functioned as the causative agents for central pathogenic cause of AD and HIV/AIDS, respectively. While both Aβ and SEVI have different genotypes/sequences, native structures, and biological functions, they also share some remarkably structural, kinetic, and cytotoxic characteristics, i.e., (i) Aβ monomers are not toxic to neuron cells, while SEVI monomers are ineffective in promoting viral infectivity^8^; (ii) both Aβ and SEVI show self-aggregation ability to form conformationally similar amyloid fibrils with dominant β-sheet structures (Fig. 1a); (iii) both Aβ and SEVI aggregates (not monomeric and fibrillar aggregates) have antibacterial activities; (iv) Similar inhibition strategies are applied to prevent the production and aggregation of Aβ and SEVI so as to reduce their neurotoxicity and HIV infectivity, respectively^10,11^. More importantly, from a clinical viewpoint, several lines of evidence suggest the common pathways and factors occurring in both HIV and AD. First, more than 50% of AIDS patients often suffer from HIV-induced neurocognitive disorders (HAND) with some symptoms similar to AD patients^12^. Second, HIV can promote the overexpression, accumulation, and aggregation of Aβ^13,14^, thus triggering the neuroinflammation and neurodegeneration in the brain^15,16^.

While both Aβ-AD and SEVI-HIV/AIDS systems have been extensively studied alone, few studies have been reported on a potential crosstalk between Aβ-AD and SEVI-HIV/AIDS. On the other hand, the abovementioned commonality between AD and HIV/AIDS raises a possibility of direct interactions between the two disease-causative proteins of Aβ and SEVI through a cross-seeding mechanism. Given that both Aβ and SEVI can misfold and self-aggregate into similar β-sheet fibrillar structures, it is intuitive to speculate that such common β-sheet structures would provide a structural basis to initiate the cross-seeding interactions between Aβ and SEVI via the compatible β-structure selection and association^9,17–20^. Moreover, microbial pathogens are also found to promote the production and overexpression of amyloid proteins (Aβ, SEVI, and prion)^21–23^, which serve as a double-edged sword of competition between (i) Aβ-/SEVI-induced prominent microbial/virus infection and inflammation^9,24^ and (ii) intrinsic antimicrobial activity of both Aβ and SEVI against several common microorganisms^25–29^. This indicates that both microbial pathogens and amyloid proteins could work together to form a bidirectional communication system that co-contributes to the pathogenesis of AD and AIDS via the “microbial infection hypothesis”^30–32^. Another interesting and fundamental point is the gender difference in both AD and AIDS, which have been observed, but not completely understood yet. Clinical data have showed that the older women (>65 years old) are more prone to have AD than the older man by ~10% at the same age groups^33–35^, presumably due to the higher risking factors of gene mutations, APOE-ɛ4 carrier and tau hyperphosphorylation in women brains^36,37^. Oppositely, SEVI amyloid fibrils only exist in male people. To our knowledge, no any study attempts to explain whether a potential link between Aβ-AD and SEVI-HIV/AIDS, if any, contributes to the gender difference in AD.

To explore a potential crosstalk between Aβ-AD and SEVI-HIV/AIDS at a molecular level, here we proposed to study the cross-seeding of SEVI (associated with HIV/AIDS) and Aβ (associated with AD) and examine their cross-seeding-induced modulation on Aβ aggregation and toxicity using a series of in-vitro and in-vivo experiments of thioflavin (ThT) fluorescence assay, atomic force microscopy (AFM), circular dichroism (CD) spectroscopy, cell viability assay (MTT), cytotoxicity assay (LDH), and Caenorhabditis elegans (C.elegans) worm models. Collective results confirmed our hypothesis that SEVI can cross-seed with Aβ with extremely high binding affinity, in which such effective cross-seeding renders SEVI as an Aβ inhibitor, as evidenced by the complete inhibition of Aβ aggregation at a very small dose of SEVI (i.e., a molar ratio of SEVI:Aβ = 0.05:1), the disassembly of the performed Aβ fibrils, and the rescue of neuroblastoma cells and C.elegans worms from Aβ-induced toxicity. More importantly, the cross-seeding-induced Aβ inhibition by SEVI could explain the gender difference in AD (i.e., AD affects more women than men) to some extent^38^, because SEIV presented only in male reduce the aggregation and spreading of Aβ in the brain.

## Results and discussion

### SEVI interacts with monomeric Aβ to inhibit amyloid aggregation

Considering that both SEVI and Aβ can form conformationally similar fibrils with common β-structures, we first investigated the cross-seeding between SEVI and Aβ, both of which are freshly co-incubated at 37 °C for 24 h with different molar ratios of SEVI (0.1–5 μM):Aβ (20 μM). ThT kinetic profiles in Fig. 1b showed that SEVI (0.1–5 μM) can effectively inhibit the aggregation of Aβ (20 μM) at sub-stoichiometric concentrations (≤equimolar ratio) in a dose-dependent manner. Specifically, a very small amount of SEVI of 0.1 μM can sufficiently reduce Aβ aggregation by 25% (brown line). Increase of SEVI concentration to 1 μM or above can almost completely eliminate Aβ aggregation as evidenced by low, steady, non-increasing ThT intensity (green & blue line). AFM images of these co-incubated Aβ-SEVI aggregates at 24 h confirmed the strong inhibitory effect of SEVI on Aβ aggregation (Fig. 1c and Supplementary Fig. 1). As compared to pure Aβ fibrils with the average height/length of 5/615 nm, the introduction of SEVI to a freshly prepared Aβ solution significantly eliminated amyloid fibril formation, instead formed some small amorphous aggregates of 40–200 nm at SEVI of 5 μM and very few observable aggregates at SEVI of 0.1 μM. All of Aβ-SEVI co-incubation systems remained in an intermediate phase of amyloid aggregation, consistent with dramatic signal drops of ThT fluorescence in a dose-dependent manner. Far-UV CD spectroscopy revealed that (i) pure SEVI (0.1–20 μM) retained random coil structures during 48 h incubation (Supplementary Fig. 2); (ii) pure Aβ displayed typical time dependent, self-aggregation bands, starting from random coil structures (negative/blue peak at 198 nm) at 0 h and ending with β-sheet structures (positive/red peak at 195 nm & negative/blue peak at 215 nm) at 24 h; (iii) in sharp contrast, co-incubation of SEVI (0.1–5 μM) with Aβ significantly delayed the structural transition of amyloid aggregates from random structures to β-sheet structures in a dose manner. Specifically, all of Aβ-SEVI aggregates retained their random coil structures up to 12 h co-incubation (Supplementary Fig. 3) and then slowly converted into less-populated β-sheet structures at 24 h (Fig. 1d). Further analysis of the secondary structure distributions from CD spectroscopy revealed that the presence of SEVI reduced both β-sheet structures by 3.3–10.4% and α-helical structures by 6.6–13.5% at the expense of increasing random coils by 9.9–24.8% (Fig. 1e).

Taken together, ThT, AFM, and CD data consistently demonstrate the cross-seeding of Aβ with SEVI, which further induces concentration-dependent inhibition effect on Aβ fibrillization by delaying the conformational transition from random coils to β-sheets and preventing amyloid growth from small amorphous aggregates to mature protofibrils/fibrils. Of note, given normal SEVI concentration in semen is 35 μg/mL^9^ (i.e., 8.75 μM), SEVI as low as 1 μM demonstrates its superior inhibition ability to completely abolish Aβ aggregation in vitro.

### SEVI interacts with different Aβ seeds to modulate the cross-seeding pathways

**Fig. 2:**
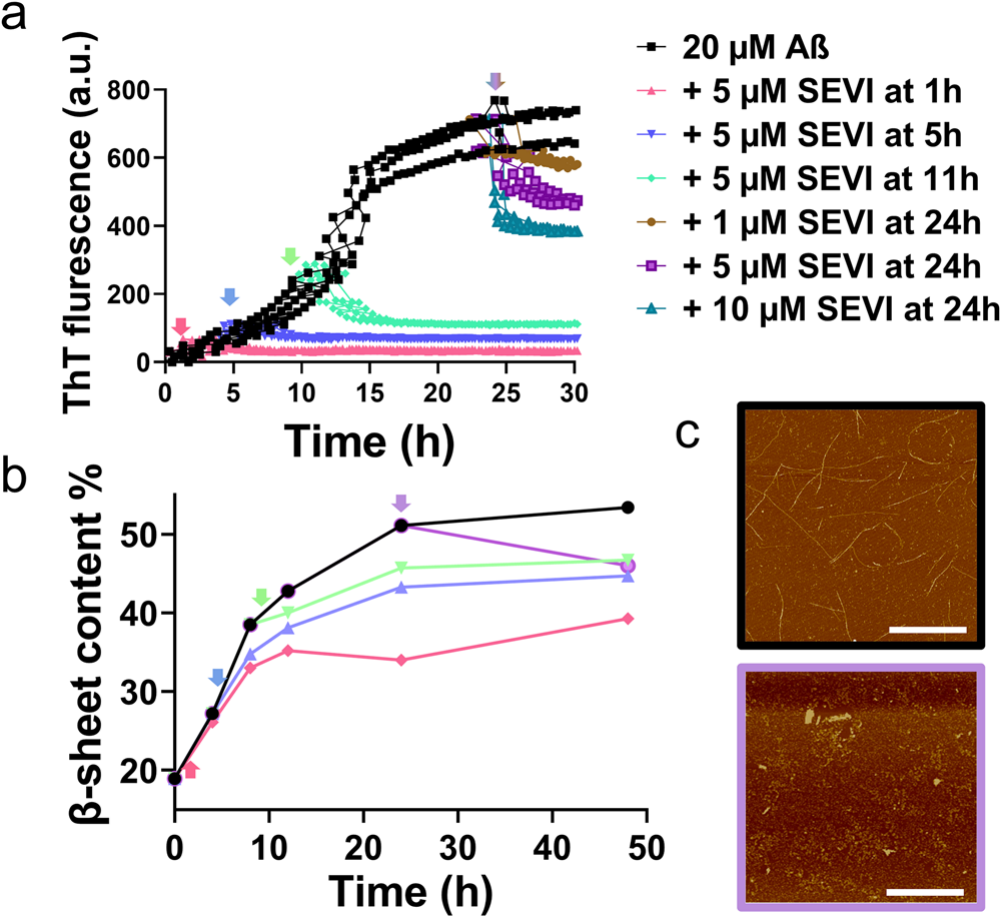
Cross-seeding of SEVI with different preformed Aβ seeds to disaggregate Aβ aggregates. Addition of SEVI (1–10 μM) to different Aβ seed solutions (20 μM) preformed at different aggregation phases of 1, 5, 11 h, and 24 h (as indicated by arrows) to show (**a**) the inhibition and disaggregation effects of SEVI on Aβ aggregates by ThT assay, all data represent mean ± standard error of triplicate measurements (*n* = 3), (**b**) the change of β-sheet structures by CD, and (**c**) the morphology of pure Aβ fibrils (black box) and the disaggregated Aβ fibrils by 5 μM SEVI (purple box) by AFM. Error bars are 1 μm.

Upon demonstrating the cross-seeding of SEVI with freshly prepared Aβ monomers to induce the cross-seeding inhibition of Aβ fibrilization, it is equally and fundamentally important to further examine whether SEVI could cross-seed with Aβ seeds preformed at 1 h of lag, 5 h of early growth, 11 h of middle growth, and 24 h of equilibrium phases to exert similar inhibition effect on Aβ aggregation. ThT aggregation curves in Fig. 2a clearly showed that when adding SEVI (5 µM) to different preformed Aβ seeds, SEVI enabled to retain its inhibition ability to prevent the further aggregation of Aβ seeds towards fibrils at every stage, as evidenced by an immediate decrease of ThT intensity at different adding points. Quantitively, comparison of the ThT data before and after adding SEVI to 1h-, 5h-, and 11h-seeded Aβ solutions, SEVI dramatically reduced final Aβ fibrillization by 95.4% (red line), 90.3% (blue line), and 83.9% (green line), respectively, as compared with pure Aβ fibrillization. Most impressively, SEVI can also disassemble the preformed Aβ fibrils obtained at 24 h and the disaggregation ability of SEVI on Aβ fibrils was dose-dependent, i.e., the increase of SEVI concentration from 1 to 10 μM led to the larger reduction of Aβ fibrillization from 12.7% (brown line) to 44.2% (cyan line). Such disassembly property of SEVI could be used as important therapeutic strategy to treat AD by the removal or remodeling of the existing Aβ fibrils.

The parallel cross-seeding experiments by CD and AFM were also conducted to better understand the structure-aggregation relationship of SEVI-Aβ cross-seeding systems. As shown in Supplementary Fig. 4, the cross-seeding of SEVI (5 μM) with different preformed Aβ seeds (i.e., 1, 5, and 11 h) led to the decreased CD spectra shift toward the non-β-sheet structures, as indicated by the reduction of peak intensities at 195 nm and 215 nm. Quantitively, as compared with pure Aβ aggregation, the addition of SEVI resulted in 14.1% (red line), 8.7% (blue line), and 6.7% (green line) β-sheet reduction in 1h-, 5h-, and 11-seeded Aβ solutions, respectively (Fig. 2b), suggesting that the existence of strong SEVI-Aβ interactions competitively interfere with Aβ structural transition, block Aβ-Aβ interactions, and prevent Aβ fibrillation. The amyloid disaggregation capacity of SEVI also depends on Aβ seeds. There exists a binding competition between cross-seeding-induced β-structure increase and disaggregation-induced β-structure decrease, i.e., despite SEVI-induced Aβ inhibition, cross-seeding still slowly and continuously increased β-sheet content before the equilibrium state, in competition with the dramatic reduction of β-sheet content upon SEVI-induced Aβ disaggregation. Additional evidences from AFM images (Fig. 2c and Supplementary Fig. 4) also confirmed that upon incubation of Aβ fibrils with 5 μM SEVI, the preformed fibrils were either significantly truncated into very thin and short fibrils of ~300 nm length, large amorphous aggregates of 150–400 nm, or small spherical particles of 20–50 nm. This disassembling, rather than inhibition effect became more obvious when adding SEVI (5 μM) into older Aβ species, as indicated by more small particles truncated from mature fibrils.

### Cross-seeding of SEVI with Aβ alleviates amyloid-mediated cytotoxicity

**Fig. 3:**
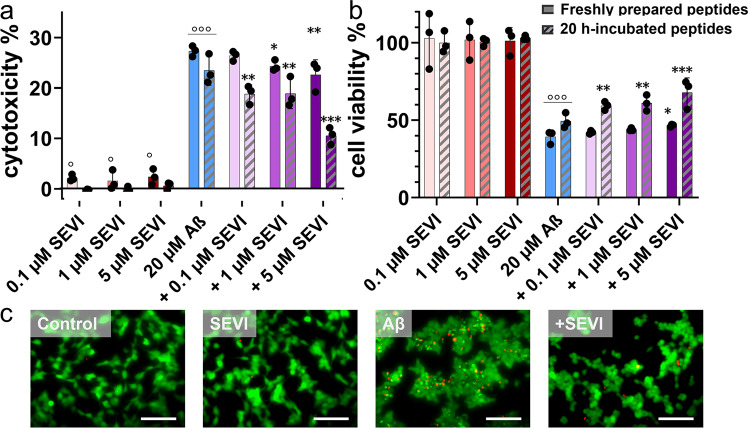
Cross-seeding of SEVI with Aβ protects cells from Aβ-induced toxicity. **a** cell cytotoxicity from LDH assay and (**b**) cell viability from MTT assay when the incubation of Aβ (20 μM) with and without SEVI at molar ratios of 1:005, 1:05 and 1:0.25 for 24 h. Untreated cells were set as control (100% MTT reduction and 0% LDH activity), cells treated with Triton-X-100 were set for 100% LDH activity. All data represent mean ± s.d. of three independent experiments. Statistical analysis (*n* = 3) was conducted for cells with both SEVI and Aβ relative to untreated cells (°*p* < 0.05; °°*p* < 0.01; °°°*p* < 0.001) or cells treated with Aβ alone (*p < 0.05; **p < 0.01; ***p < 0.001). **c** Representative fluorescence microscopy images of cells treated for 24 h with 20 h-incubated Aβ (20 µM) in the absence or presence of 5 µM SEVI. Untreated cells were set as control. Red and green fluorescence indicate the dead and live cells, respectively. Scale bars are 100 µm.

The inhibition of amyloid fibrillization by any inhibitor does not necessarily indicate that this inhibitor would also reduce amyloid-induced toxicity, simply because (i) amyloid oligomerization and fibrillation could be the two different pathological pathways and (ii) inhibitor-amyloid complexes or inhibitor-induced amyloid oligomers could be either on- or off-pathway species. Here, we further examined whether SEVI can protect human neuroblastoma SH-SY5Y cells from Aβ-induced toxicity using LDH (Fig. 3a), MTT (Fig. 3b), and live/dead assays (Fig. 3c). As a control, pure SEVI of different concentrations (0.1–5 μM) were almost non-toxic to SH-SY5Y cells (99.7–101.3% cell viability and 1.6–2.4% cytotoxicity), in sharp contrast to highly toxic Aβ (20 μM) with cell viability of 39.3% and cell apoptosis of 27.6%. When co-culturing SEVI with Aβ-treated SH-SY5Y cells, SEVI exhibited a concentration-dependent cell protection, as indicated by an increase in cell viability by 2.6–7.4% and a reduction in cytotoxicity by 1.1–5.0% as SEVI concentration increased from 0.1 to 5 μM. It appears that SEVI exhibits the limited protection effect on Aβ-induced cell toxicity, which could be attributed to the less inhibition on the formation of (i) toxic Aβ aggregates and (ii) less- or none-toxic SEVI-Aβ complexes. More fundamentally, both Aβ oligomers and SEVI-Aβ complexes might have a wide structural and functional diversity along the complex cross-seeding pathways. Some might be extremely toxic to initiate the apoptosis pathways, while the others are possibly off-pathway species that proceed to the fibril formation with less cell toxicity.

Apart from incubating cells with freshly prepared SEVI and Aβ together, we also designed a set of pre-incubation experiments by incubating Aβ with and without SEVI at 37 °C for 20 h before introducing them into cells. Side-by-side comparisons between the two types of simultaneous incubation and pre-incubation tests in Fig. 3a, b revealed some similarities and differences. First, similar to simultaneous incubation tests, pre-incubation tests showed that pure SEVI of 0.1–5 μM alone did not introduce any toxicity to cells, while preformed Aβ fibrils (20 μM) were highly toxic to cells, leading to 48.4% of cell viability and 23.5% of cell cytotoxicity. Second, for all of cross-seeding cases, the pre-incubation tests (shadow bars) showed the lower cell toxicity and the higher cell viability than the corresponding simultaneous incubation tests (solid bars). This again indicates that the cross-seeding of SEVI with Aβ in cell medium solution allows to be more effective to form less toxic species or remove more toxic species than those cross-seeding species in cultured cells. As a result, upon introducing cross-seeding species into cells, cell viability increased to 59.4%, 61.1%, and 67.9%, while cell apoptosis decreased to 18.8%, 18.8%, and 10.1% at Aβ:SEVI ratios of 1:0.005, 1:0.05, and 1:0.25, respectively. Furthermore, seeing is believing. Live/dead cell assay confirmed (i) the non-toxicity of pure SEVI and (ii) the protection ability of SEVI on Aβ-induced cell death (Fig. 3c).

### Cross-seeding of SEVI with Aβ rescues Aβ-mediated dysfunction in worm models

**Fig. 4:**
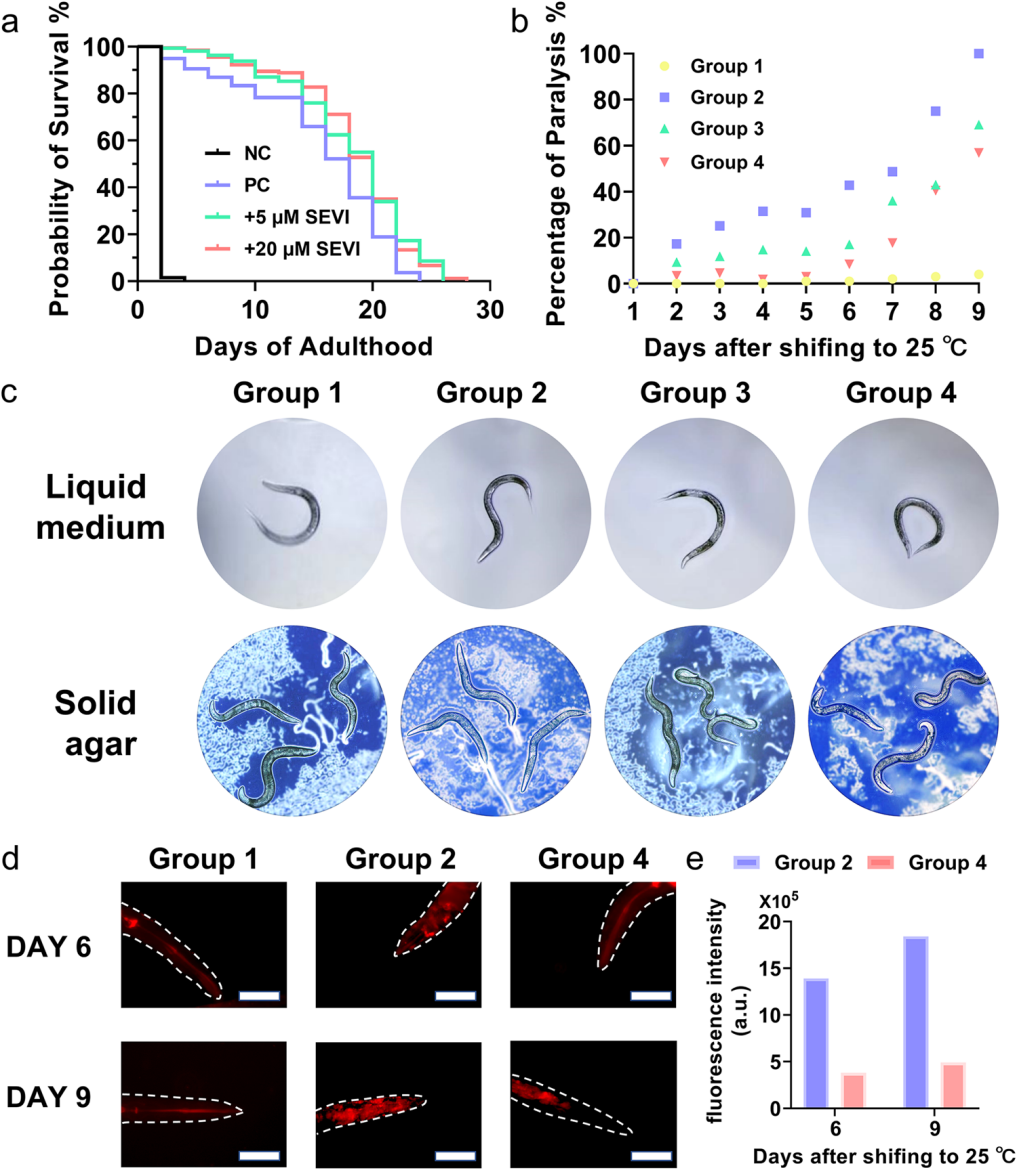
SEVI reduces Aβ-induced paralysis in transgenic AD C. elegans. **a** Lifespan recording of ~120 age-synchronized N2 worms/group incubated with and without S-complete buffer (blue), 1 M NaOH (black), 5 μM SEVI (green), and 20 μM SEVI (red). Paralysis assays for characterizing (**b**) paralysis recording, (**c**) paralysis state in liquid medium (top) and on agar plates (bottom), (**d**) representative in vivo NIAD-4 staining, and (**e**) corresponding Aβ fluorescence intensity of age-synchronized GMC101 worms/group incubated in the absence (group 2) or presence of 5 μM SEVI (group 3) and 20 µM SEVI (group 4) at 20 °C (group 1) or 25 °C (groups 2–4) for 9 days. Scale bars are 100 µm.

To make this fundamental study more clinically relevant, we used Caenorhabditis elegans model including (i) wild type C. elegans N2 strain for lifespan assay to test if SEVI has any toxic effect on C. elegans and (ii) transgenic C. elegans GMC101 strain that express human Aβ_1–42_ for paralysis assay to investigate the potential neuroprotective effects of SEVI on Aβ-induced toxicity. As shown in Fig. 4a, to establish benchmarks, a negative control of 1 M NaOH caused 100% worm death after 4 days, while a positive control of S complete buffer-DMSO experienced a normal survival life cycle of being dead in 2–3 weeks. For comparison, the SEVI-treated N2 worms (5 and 20 μM of SEVI) did not show any SEVI-induced toxicity, instead prolonged their lifespan by additional 48–96 h, as indicated by the delayed worm mortality of the SEVI-treated group (green and red) compared with the control group (black and blue).

Upon demonstrating that SEVI and its dosage are non-toxic to the life cycle of worms, we then introduced the transgenic, age-synchronized C. elegans GMC101 strain (namely, Aβ worms) to examine whether supplementation of SEVI in Aβ worms may affect the progression of paralysis as induced by Aβ toxicity. This GMC 101 strain shows a phenotype of Aβ_1–42_ expression and aggregation in body wall muscles after temperature upshifting from 20 °C to 25 °C, leading to age-dependent progressive paralysis. Aβ worms with or without 5–20 μM of SEVI treatment were separated into four groups for testing paralysis progression before and after temperature upshifting: (1) Aβ worms cultured in 20 °C that are not expected to express any Aβ, (2) Aβ worms cultured in 25 °C alone, (3) Aβ worms cultured in 25 °C with 5 μM SEVI as supplementary food, and (4) Aβ worms cultured in 25 °C with 20 μM SEVI as supplementary food. Here, we define three mobility states to describe the paralysis of Aβ worms, i.e., (i) Self-Moving (SM): worms can move > 1 body length by themselves; (ii) Touch-Quick (TQ): worms can move > 1 body length upon immediate touch; (iii) Touch-Slow (TS, paralysis): worms unable to react or move < 1 body length after touch. All of these mobility behaviors of Aβ worms were recorded and analyzed for 9 days after temperature upshifting from 20 to 25 °C (Supplementary Fig. 5). As a control, the untreated worms cultured at 20 °C in the group 1 did not produce any paralysis. In sharp contrast, the untreated worms cultured at 25 °C in the group 2 showed a typical time dependent, progressive paralysis, as evidenced by 41%/41%/17% of SM/TQ/TS behavior within 48 h, 0%/51%/48% of SM/TQ/TS behavior within 168 h, and complete paralysis within 216 h (Fig. 4b and Supplementary Fig. 5). For comparison, within the same 9 days that cause the complete paralysis of Aβ-accumulated worms in the group 2, worms treated with SEVI of 5 μM (group 3) or 20 μM (group 4) largely decreased their paralysis rate to 69.1% and 56.8%, respectively. Evidently, the higher concentration of SEVI (20 μM) is more effective for delaying worm paralysis than the lower concentrations of SEVI (5 μM). Seeing is believing. In Supplementary Movie 1–2 and Fig. 4c, representative worms of group 1–4 obtained from day 9 showed that (i) the untreated worms cultured in 20 °C (yellow) and 25 °C (blue) displayed very different mobility behaviors including the bending rate, bending degree, crawl/swim speed, and body shape; (ii) after 9 days incubation at 25 °C, Aβ worms (blue) as a control group were almost completely paralysis as evidenced by no any movement and rigid “rod-like” shape. Differently, SEVI treatment by 5 μM (green) or 20 μM (red) can effectively suppress worm paralysis by protecting the worms from Aβ-induced toxicity. Consistently, the amount of Aβ accumulation in their head region at day 6 and 9, as stained by Aβ-specific dye NIAD-4, showed large differences between three groups of 1, 2, and 4 (Fig. 4d and Supplementary Fig. 5). Specifically, the worms in the group 1, as incubated at 20 °C, did not express Aβ, i.e., no Aβ deposition was observed due to the absence of fluorescence stain (red area) in worm body (dash line in Fig. 4d). Differently, upon temperature upshifting from 20 °C to 25 °C, the worms in the group 2 showed a significant increase in Aβ deposition (red) in their head regions. As compared to the worms in the group 2, the worms treated with SEVI (20 μM) in the group 4 showed even more reduction in Aβ deposits (red fluorescence) by 72.6% at day 6 and 73.3% at day 9 (Fig. 4e). Overall, these findings from transgenic C. elegan worm model confirm that SEVI attenuates Aβ-induced toxicity, delay Aβ-induced paralysis in the C. elegans, and extend the lifespan of C. elegans.

## Conclusions

Identification and understanding of the cross-seeding interactions between different disease- causative proteins are critical for both pathophysiological process of individual diseases and pathophysiological crosstalk between diseases. In this study, we demonstrate the cross-seeding of SEVI (associated with HIV/AIDS) with Aβ (associated with AD) using a combination of spectroscopy approaches, cell assays, and transgenic worm models. The resultant cross-seeding results from ThT, AFM, and CD confirmed that SEVI of even 1 μM can cross-seed with (i) both Aβ monomers and oligomers to completely inhibit fibril formation and (ii) Aβ fibrils to disassemble these preformed fibrils into small amorphous aggregates. Such cross-seeding-induced Aβ inhibition by SEVI is also contributed by the delay or reverse of conformational transition of Aβ aggregates towards β-sheet structures. Consequently, the strong cross-seeding interactions between SEVI and Aβ enabled to protect SH-SY5Y neurons and transgenic C. elegans from Aβ-induced toxicity, as evidenced by increasing 2.6–19.5% of cell viability, reducing of 1.1–13.4% cell cytotoxicity, suppressing 43.2% of worm paralysis, and extending 16.7% of worm lifespan. More importantly, considering the basic facts that (i) Women have more AD than men in the same age groups and (ii) SEVI is only presented in men and thus serve as a key gender marker to distinguish men from women, the cross-seeding between SEVI and Aβ indicates that SEVI can function as an effective defense agent (i.e., Aβ inhibitor) to prevent the pathological aggregation of Aβ and thus to protect men from Aβ-induced AD, which could be considered as one of factors (e.g., genetic, biological, lifestyle and societal factors) to explain the gender difference in AD. As always, more fundamental research and clinical trials are indeed warranted to discover the pathological link and functions between Aβ-AD and SEVI-HIV/AIDS.

Given by the close relationship between Alzheimer disease (AD) and Type II diabetes (T2D) at a clinical level, we further to examine the possible cross-seeding interactions between SEVI and hIAPP (associate with T2D). Unfortunately, our preliminary ThT data showed that no obvious cross-seeding occurred between SEVI and hIAPP at three different SEVI:hIAPP ratios of 1:2, 1:1, and 2:1 (Supplementary Fig. 6), probably because of unfavorable electrostatic repulsion between two positively charged SEVI and hIAPP peptides. To the best of our knowledge, there have been few reports on the cross-seeding interactions between SEVI and amyloid peptides before. We are currently still exploring the pathological networks between SEVI and other disease-related proteins and more work toward this goal is required.

## Methods

### Reagents

Amyloid beta (Aβ_1–42_, ≥ 95%) was purchased from CPC Scientific (Sunnyvale, CA). Synthetic SEVI was obtained from Genscript (Piscataway, NJ). 1,1,1,3,3,3-hexafluoro-2-propanol (HFIP, ≥ 99.9%), dimethyl sulfoxide (DMSO, ≥ 99.9%), and thioflavin T (ThT, 98%) were commercially available from Sigma Aldrich (St. Louis, MO). All other chemicals were of the highest grade available.

### Peptide purification and preparation

Both Aβ and SEVI were stored at −20 °C immediately once received. To obtain monomeric Aβ and SEVI, 1 mg lyophilized peptide was then redissolved in 1 mL HFIP for 2 h at room temperature, followed by 30 min sonication in ice bath, 30 min centrifugation at 14,000 rpm, and 4 °C. Supernatant was then extracted and distributed into different packages according to experiment needs. Sub-packages were frozen in −80 °C before use. Unless otherwise mentioned, all the peptides were first dried with freeze-dryer, pre-dissolved in 10 mM NaOH at the concentration of 2 μg/μL and then further dissolved in buffer solution.

### Thioflavin T (ThT) fluorescence assay

Amyloidosis kinetics of Aβ was monitored by using ThT fluorescence assay. To determine the effect of SEVI on monomeric Aβ, samples were prepared on ice by mixing freshly prepared Aβ was diluted into SEVI samples or buffer containing 5 μM ThT to prepare Aβ solution at 20 μM. The aggregation kinetics was then initiated at 37 °C and fluorescence intensity data were recorded consistently at 30 min intervals for 24 h. For seeding experiment, 5 μL SEVI-10mM NaOH mixture were added into aggregating Aβ solution at 1, 5, 11, and 24 h and kept monitor aggregation curves to illustrate the cross-seeding between SEVI and seeded Aβ. The kinetic top-read mode of a SpectraMax M3 microplate reader (Molecular Devices, CA, USA) with excitation at 450 nm and emission at the range of 470 nm to 500 nm was used to monitor the ThT fluorescence.

### Circular dichroism spectroscopy (CD)

The secondary structures of peptides were examined by far-UV CD spectroscopy with a J-1500 spectropolarimeter (Jasco Inc., Japan) using a continuous scanning mode at room temperature. The time-course samples collected for CD measurement were prepared in the same condition as for the ThT assay except using PBS buffer (pH = 7.4). At each time point (e.g., 0, 4, 8, 12, 24, and 48 h), 150 μL sample solution was placed into a 1 mm optical path length CD cuvette and spectrum was collected from 250 to 190 nm with a step size of 0.5 nm and 50 nm/min scan rate. All the treatment groups were tested for three times and further corrected by subtracting PBS buffer baseline to remove background influence. The secondary structural contents were determined by using the Beta Structure Selection (BeStSel) algorithm^39^ (http://bestsel.elte.hu/).

### Atomic force microscopy (AFM)

AFM was performed using Nanoscope III multimode AFM with an Extender eletroncis module (Veeco Corp, Santa Barbara, CA) in a ScanAsyst Mode. 20 μL of the samples were deposited on a freshly cleaved mica sheet. After 5 min, the sample was washed three times using Mill-Q water to remove salts and dried with air gas. The cantilever resonance frequency was 45–95 kHz. The images (256 pixels × 256 pixels) were captured using a scan size of 3 μm. Representative AFM images were obtained by scanning six different locations on the mica surface.

### Cell culture

Neuroblastoma cell line SH-SY5Y (ATCC® CRL-2266, Manassas, VA) was selected to investigate the protection ability of SEVI on Aβ induced cell damage. SH-SY5Y cell lines were maintained in DMEM (Sigma Aldrich, St. Louis, MO) supplemented with 10% (v/v) fetal bovine serum (VWR, Radnor, PA) and 1% (v/v) penicillin/streptomycin (Sigma Aldrich, St. Louis, MO) in a T75 flask in humidified air containing 5% CO2 at 37 °C. After cells grown to ~80% confluent, they were then harvested by using 0.25 mg/mL Trypsin/EDTA solution (Sigma Aldrich, St. Louis, MO) and seeded in 96-well plate (2 × 10^4^ per well).

### MTT reduction assay

A colorimetric MTT metabolic activity assay was used to determine cell viability. SH-SY5Y cells (2 × 10^4^ cells/well) were cultured in a 96-well plate at 37 °C overnight. Then replace with fresh cell culture medium with freshly prepared or 20 h pre-incubated Aβ/SEVI/Aβ-SEVI solutions and cultured for another 24 h at 37 °C and 5% CO_2_ in a humidified incubator. After 24 h, all suspension liquid was removed, replaced by MTT reagent (Invitrogen, Carlsbad, CA) and incubated for another 4 h at 37 °C. Finally, the resultant formazan crystals were dissolved in dimethyl sulfoxide and the absorbance was measured at 540 nm using a microplate reader. Each sample repeated as least three times and cell viability were calculated in comparison with untreated cells.

### Lactate dehydrogenase (LDH) cytotoxicity assay

The amyloid-induced cytotoxicity was further evaluated by LDH assay. LDH release from cytoplasm to medium caused by membrane leakage was measured as biomarker to quantify the cytotoxicity. LDH assay was performed using CytoSelect^TM^ LDH Cytotoxicity Assay Kit (Cell Biolabs, San Diego, USA). Briefly, sterile water and Triton X-100 solution was added as positive and negative control, respective and incubated for 10 min at room temperature. Then transfer 90 μL of medium from each well to a clean 96-well plate, followed by adding 10 μL LDH Cytotoxicity Assay Reagent, and incubated for another 30 min in dark area. Absorbance was read at the wavelength of 450 nm by microplate reader. The results were normalized by negative and positive control as a relative cytotoxicity percentage. Data were exhibited in mean ± s.d. of three independent tests.

### LIVE/DEAD viability/cytotoxicity assay

LIVE/DEAD cell assay was further used to visualize the amyloid-mediated cell viability and cytotoxicity. Aβ incubated with and without SEVI for 24 h was stained using a LIVE/DEAD® Viability/Cytotoxicity Kit (L3224, Invitrogen) and imaged by fluorescence microscope (Olympus IX81) to visualize the live and dead insulinoma cells. Briefly, 100 μL staining solution containing 0.05 μL calcein AM and 0.2 μL ethidium homodimer-1. The representative images of the live and dead cells were chosen from three different locations.

### C.elegans experiments

C. elegans strains used in this study (i) dvIs100[unc-54p::A-beta-1–42::unc543′UTR + mtl-2p::GFP] (GMC101) and (ii) wild-type (N2) were provided by the Caenorhabditis Genetics Center (University of Minnesota). C. elegans strains cultured at 20 °C on nematode growth medium (NGM) agar plates seeded with E. coli strain (OP50) unless stated otherwise. Saturated cultures of OP50 were grown by inoculating 20 ml of LB medium with OP50 and incubating the culture overnight at 37 °C and stored at 4 °C for further use. NGM plates were seeded with bacteria by adding 150 μL of saturated OP50 to each plate (10 cm plate) and leaving the plates dried at room temperature for 1 day. Hypochlorite bleaching was then performed on a plate full of eggs and gravid adults to establish a synchronous population, followed by hatching overnight in S-complete buffer and cultured on NGM Agar plates or liquid medium seeded with the E. coli strain OP50^40^. After reach to L4 larval stage, 5-fluoro-2′-deoxyuridine (FudR) was added to inhibit growth of offspring.

### Wild-type C.elegans lifespan assay

Lifespan assay was modified slightly according to the methods previously described^41^. Briefly, after establishing a synchronous population, worms/OP50 solution was transferred to a 96 well plate. For each group, at least 120 worms were counted. Sealed the plate to avoid contamination and evaporation and allowed worms to grow up to L4 stage. 30 μL of a 0.6 mM FUDR stock solution was then added to each well. 5 μL SEVI solution prepared in S-complete buffer containing < 0.1% DMSO at an appropriate concentration (5 μM/20 μM) was then added to each well. As controls, the same amount of S-complete buffer-DMSO or 1 M NaOH as positive and negative control respectively was added to each well. After adding drugs, the number of survival worms was scored under the microscope at 2 days intervals until the last worm dead.

### C.elegans motility assay

3.5 cm NGM Agar plate (including 75 μM FudR) was prepared by adding 100 μL OD50 and 120 μL tested drugs. SEVI solution (5 μM/20 μM) or control was prepared in sterile water containing < 0.1% DMSO. Tested solution was distributed over the entire plate surface and allowed to dry in a sterile hood with the lid open for at least 1 h. Plates were then allowed to sit at 20 °C for 24 h before use.

Age-synchronized worms were grown at 20 °C until the L4 larval stage and then transferred to fresh 3.5 cm NGM Agar plates treated with Control-DMSO plates or SEVI. For each group, three individual plates were tested, and each plate contained ~30 worms. Plates were immediately shifted to 25 °C and paralysis was scored 24 h after the temperature shift. Animals were scored as paralyzed if they failed to move during observation and failed to respond to touch-provoked movement with a platinum wire. Representative worms were transferred to liquid M9 buffer (1 worm) or Agar plate (3 worms) for further photo taking and video recording.

### Staining and microscopy in living C. elegans

20 worms were randomly selected from different times and drug/control treated plates and incubation with 1 μM NIAD-4 (0.1% DMSO in M9 buffer) for 4 h at room temperature gave robust and reproducible staining. Worms/NIAD solution was then transferred on 2% agarose pads containing 40 mM NaN3 as anaesthetic on glass microscope slides for imaging. Images were captured by fluorescence microscope (Olympus IX81) with × 40 objective and a CY3/DAPI filter. Fluorescence intensity was calculated using ImageJ software and normalized as the corrected total cell fluorescence. Only the head region was considered because of the high background signal in the guts.

### Statistics and reproducibility

In vitro treatments including ThT assay and cell assay contain at least 3 independent replicates. Statistical significance between the groups in Fig. 2a, b were evaluated using one-way ANOVA test in GraphPad. The sample sizes of C.elegan worm experiments are indicated in the main text and figure legends.

### Reporting summary

Further information on research design is available in the Nature Research Reporting Summary linked to this article.

## Supplementary information

Supplementary Information

Description of Additional Supplementary Files

Supplementary Movie 1

Supplementary Movie 2

Supplementary Data 1

Reporting Summary

## Data Availability

The data that support the findings of this study are available from the corresponding author and co-authors upon reasonable request. All relevant data for the figures are included in the supplementary information files. Source data has been provided as Supplementary Data 1.
