# Supplementary material for: Cross-seeding between Aβ and SEVI indicates a pathogenic link and gender difference between alzheimer diseases and AIDS
Source: Commun Biol. 2022 May 5;5:417. doi: 10.1038/s42003-022-03343-7 (PMC9072343; doi:10.1038/s42003-022-03343-7)
Supplement: Supplementary file 1 — Supplementary Information [file 42003_2022_3343_MOESM1_ESM.pdf]

Supplemental Materials

Cross-seeding between Aβ and SEVI Indicates a Pathogenic Link and Gender Difference between Alzheimer Diseases and AIDS

Yijing Tang[1], Dong Zhang[1], Yanxian Zhang[1], Yonglan Liu[1], Yifat Miller[2], Keven Gong[3], and Jie Zheng[1*]

[1]Department of Chemical, Biomolecular, and Corrosion Engineering
The University of Akron, Ohio, USA, 44325

[2]Department of Chemistry
Ben-Gurion University of the Negev, Be'er Sheva, Israel, 84105

[3]Western Reserve Academy, Hudson, Ohio, USA, 44236

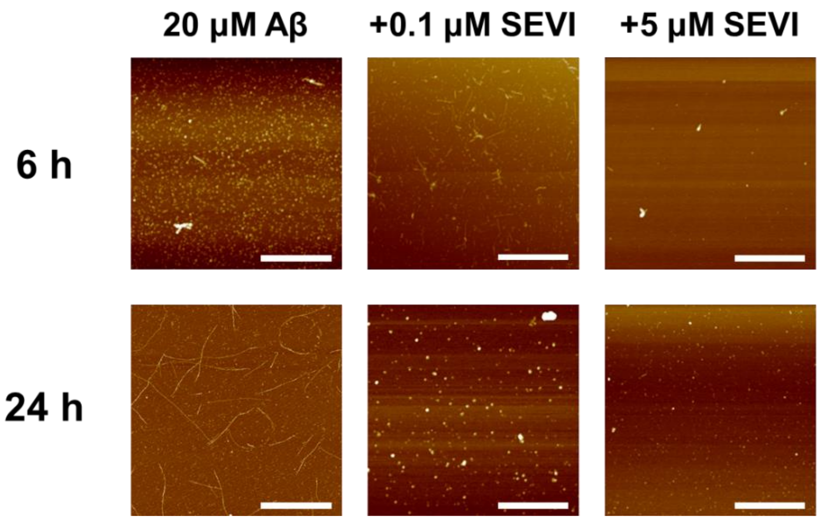

Supplementary Figure 1. Cross-seeding of SEVI with Aβ to delay fibril formation. AFM images for pure Aβ peptides (20 μM) in the absence and presence of different concentrations of SEVI (0.1 μM and 5 μM) at 6 and 24 h. Scale bars are 1 μm.

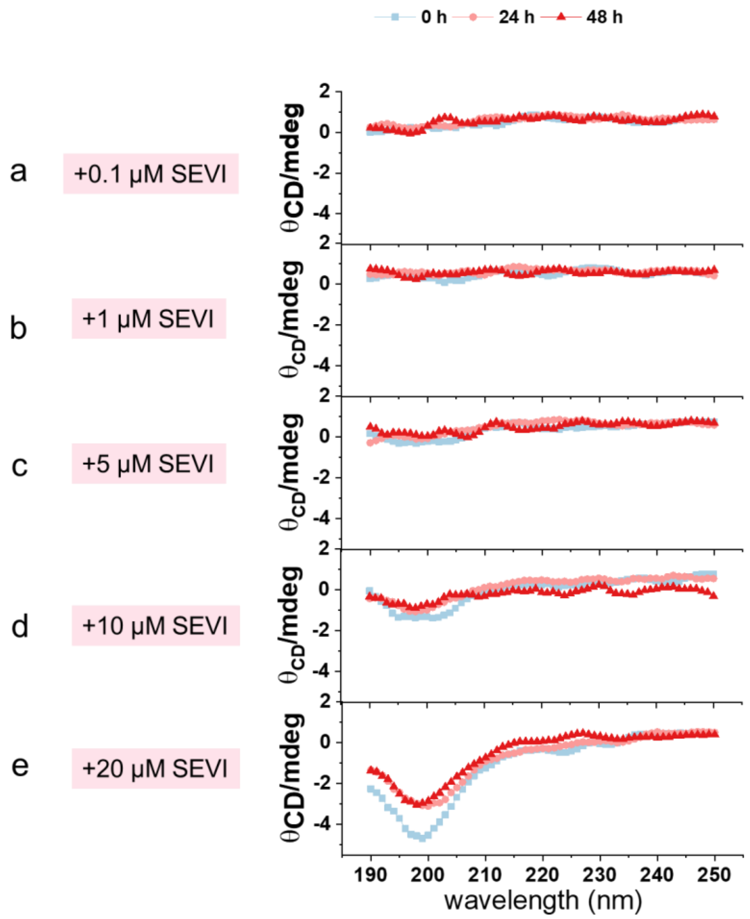

Supplementary Figure 2. Non-amyloidogenic concentrations of SEVI. Time-dependent circular dichroism (CD) spectra of SEVI of **a** 0.1 µM, **b** 1 µM, **c** 5 µM, **d** 10 µM, and **e** 20 µM.

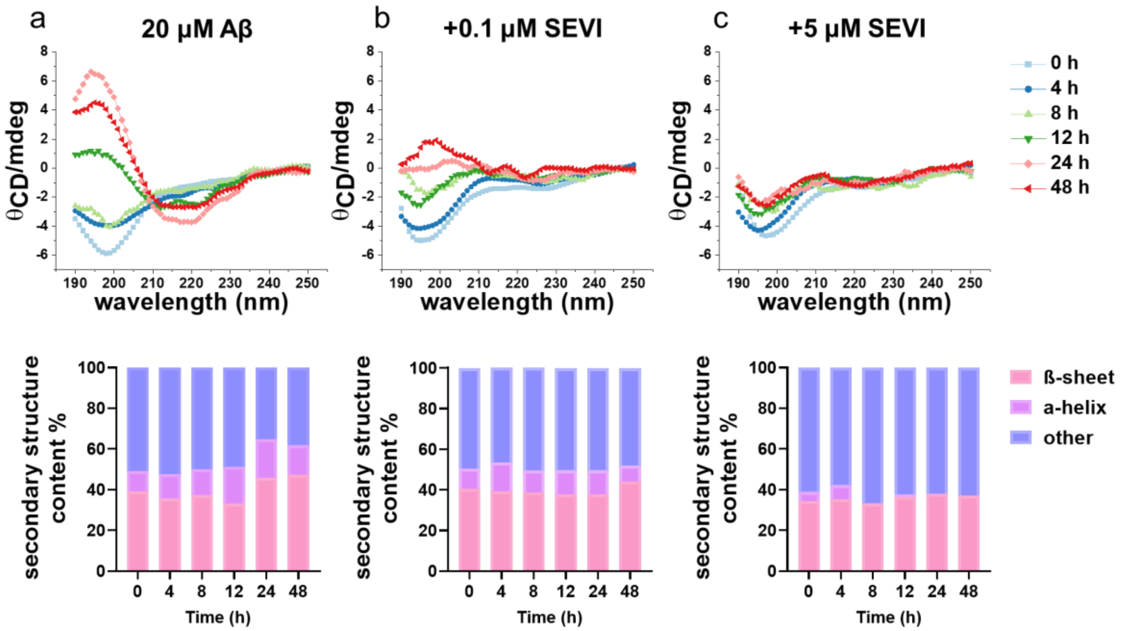

Supplementary Figure 3. Cross-seeding of SEVI with Aβ to retard secondary structure transitions. CD spectra and the corresponding secondary structure contents for **a** 20 μM Aβ in the absence and presence of **b** 0.1 μM and **c** 5 μM of SEVI during 48 h of incubation.

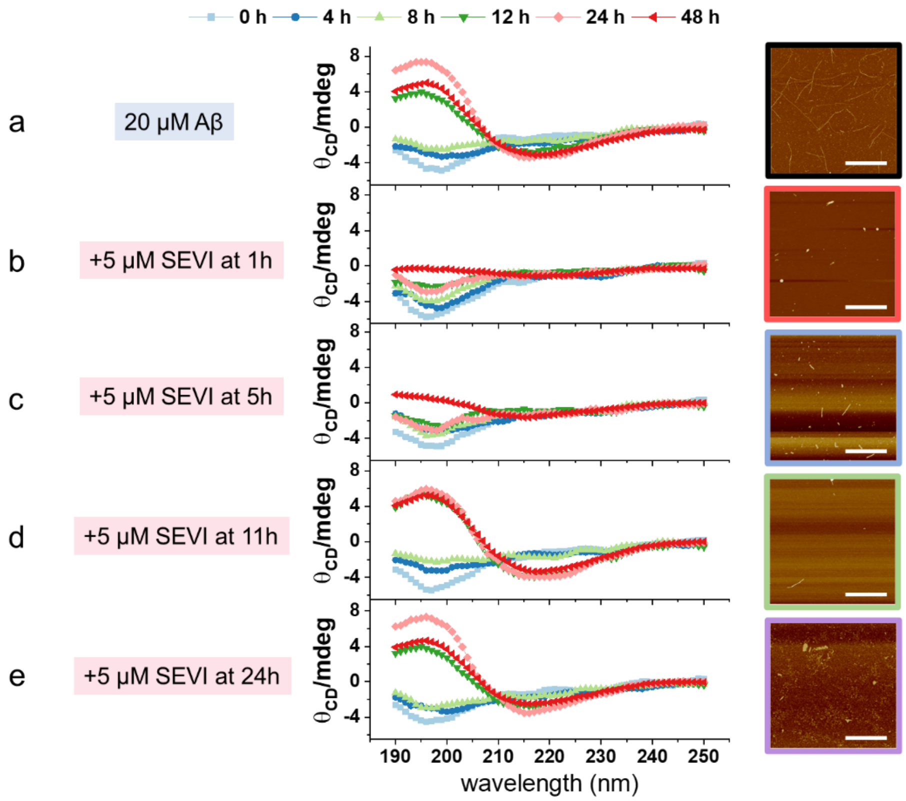

Supplementary Figure 4. Cross-seeding of SEVI with different preformed Aβ seeds to redirect amyloid formation pathways. Time-dependent CD spectra and final AFM images for characterizing the secondary structure and morphology of cross-seeding of SEVI (5 µM) with Aβ (20 µM) seeds at different time points of **a** 0, **b** 1, **c** 5, **d** 11, and **e** 24 h. Scale bars are 1 µm.

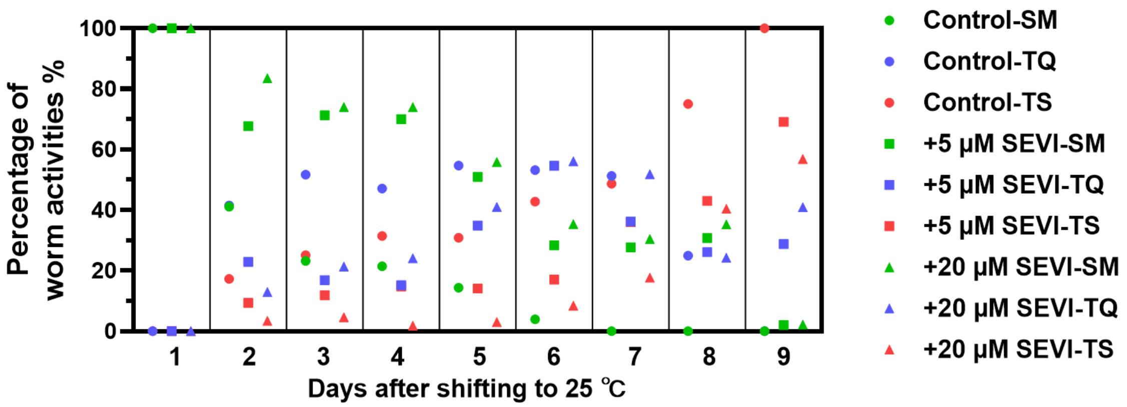

Supplementary Figure 5. SEVI reduces Aβ-induced paralysis in transgenic AD C. elegans. Concentration effect of SEVI of 5-20 μM on the moving activities of Aβ worms. Three distinct moving activities are defined by Self-Moving (SM): worms can move > 1 body length by themselves. Touch-Quick (TQ): worms can move > 1 body length after touch. Touch-Slow (TQ)/paralysis: worms can move < 1 body length after touch.

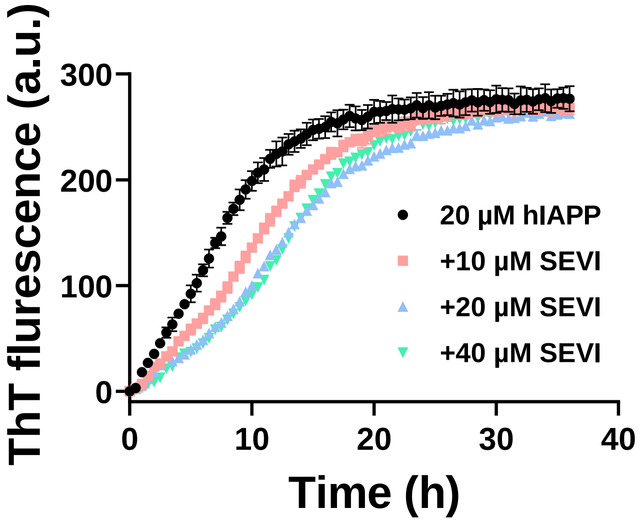

Supplementary Figure 6. SEVI unable to cross-interact with hIAPP to inhibit its aggregation. Time-dependent ThT fluorescence profiles for co-incubation of hIAPP (20 μM) with and without SEVI (10-40 μM).
