# Supplementary material for: Cross-seeding between Aβ and SEVI indicates a pathogenic link and gender difference between alzheimer diseases and AIDS
Source: Commun Biol. 2022 May 5;5:417. doi: 10.1038/s42003-022-03343-7 (PMC9072343; doi:10.1038/s42003-022-03343-7)
Supplement: Supplementary file 2 — Description of Additional Supplementary Files [file 42003_2022_3343_MOESM2_ESM.pdf]

Description of Additional Supplementary Files

File name: Supplementary Movie 1
Description: Paralysis Recording of GMC 101 in Liquid Medium.

File name: Supplementary Movie 2
Description: Paralysis Recording of GMC 101 on Agar Plate.

File name: Supplementary Data 1
Description: Source data.
